# Quantification of left coronary bifurcation angles and plaques by coronary computed tomography angiography for prediction of significant coronary stenosis: A preliminary study with dual-source CT

**DOI:** 10.1371/journal.pone.0174352

**Published:** 2017-03-27

**Authors:** Yue Cui, Wenjuan Zeng, Jie Yu, Jing Lu, Yuannan Hu, Nan Diao, Bo Liang, Ping Han, Heshui Shi

**Affiliations:** 1 Department of Radiology, Union Hospital, Tongji Medical College, Huazhong University of Science and Technology, Wuhan, China; 2 Department of Clinical Laboratory, Union Hospital, Tongji Medical College, Huazhong University of Science and Technology, Wuhan, China; 3 Department of Nuclear Medicine, Zhongshan Hospital, Xiamen University, Xiamen, Fujian, China; Universita degli Studi Magna Graecia di Catanzaro, ITALY

## Abstract

**Purpose:**

To evaluate the diagnostic performance of left coronary bifurcation angles and plaque characteristics for prediction of coronary stenosis by dual-source CT.

**Methods:**

106 patients suspected of coronary artery disease undergoing both coronary computed tomography angiography (CCTA) and invasive coronary angiography (CAG) within three months were included. Left coronary bifurcation angles including the angles between the left anterior descending artery and left circumflex artery (LAD-LCx), left main coronary artery and left anterior descending artery (LM-LAD), left main coronary artery and left circumflex artery (LM-LCx) were measured on CT images. CCTA plaque parameters were calculated by plaque analysis software. Coronary stenosis ≥ 50% by CAG was defined as significant.

**Results:**

106 patients with 318 left coronary bifurcation angles and 126 vessels were analyzed. The bifurcation angle of LAD-LCx was significantly larger in left coronary stenosis ≥ 50% than stenosis < 50%, and significantly wider in the non-calcified plaque group than calcified. Multivariable analyses showed the bifurcation angle of LAD-LCx was an independent predictor for significant left coronary stenosis (OR = 1.423, *P* = 0.002). In ROC curve analysis, LAD-LCx predicted significant left coronary stenosis with a sensitivity of 66.7%, specificity of 78.4%, positive predictive value of 85.2% and negative predictive value of 55.8%. The lipid plaque volume improved the diagnostic performance of CCTA diameter stenosis (AUC: 0.854 vs. 0.900, *P* = 0.045) in significant coronary stenosis.

**Conclusions:**

The bifurcation angle of LAD-LCx could predict significant left coronary stenosis. Wider LAD-LCx is related to non-calcified lesions. Lipid plaque volume could improve the diagnostic performance of CCTA for coronary stenosis prediction.

## Introduction

Coronary artery disease (CAD) is the leading cause for death in developed and developing countries [1]. The most common reason of CAD is atherosclerosis. The intravascular ultrasound (IVUS) is recommended as the reference standard to quantify atherosclerosis plaques [2]. However, IVUS is an invasive examination with expensive costs and high risks for the routinely clinical application. Coronary computed tomography angiography (CCTA) is a reliable non-invasive imaging modality that is widely used for the diagnosis of coronary stenosis with high sensitivity and negative predictive value [3, 4]. Additionally, CCTA can measure coronary bifurcation angles with high accuracy [5]. The three-dimensional (3D) geometry construction of coronary artery bifurcations could have an effect on the hemodynamic flow patterns and play an important role in the plaque formation, distribution and composition [6, 7]. By analyzing the hemodynamic of various left coronary bifurcation angle in simulated and realistic models, Chaichana et al. found that wider bifurcation angles might induce low wall shear stress, which may lead to the development and progression of CAD [6–8]. In addition, CCTA allows visualization and quantification of plaque characteristics [9, 10]. Coronary high-risk plaque features, including low attenuation non-calcified plaque, positive remodeling, napkin-ring sign and spotty calcium, may be associated with acute coronary syndrome (ACS), adverse cardiovascular events and ischemia [11, 12]. CCTA has the capability to evaluate and quantify the potentially high-risk plaques without extra imaging, which could improve image-guided prevention, interventions and therapy [11, 12]. However, the diagnostic performance of quantification of left coronary bifurcation angles and plaque characteristics by CCTA for prediction of significant coronary stenosis has been less extensively studied. The aim of this study was to evaluate the diagnostic performance of left coronary bifurcation angles and the CCTA plaque parameters for the prediction of significant coronary stenosis using invasive coronary angiography (CAG) as the reference standard and explore the potential risk factors for development of atherosclerosis. We hypothesized that the left coronary bifurcation angle of LAD-LCx and quantitative plaque analysis represent a more accurate method for diagnosis of significant coronary stenosis.

## Materials and methods

### Study population

Between January 2010 and March 2016, a total of 119 consecutive patients suspected of CAD who underwent CCTA and CAG within three months were retrospectively screened for the present study. Exclusion criteria were renal insufficiency with serum creatinine levels >1.5 mg/dl, contrast materials allergy, congenital coronary artery anomalies, left main coronary disease (defined as ≥ 50% luminal stenosis by CAG), poor image quality and history of coronary artery bypass graft surgery (CABG) or previous coronary stents. Clinical data were acquired by reviewing the electronic medical records of patients. This study was approved by the Ethics Committee of Tongji Medical College, Huazhong University of Science and Technology. Written informed consent was obtained from all participants.

### Coronary CT angiography protocol

Coronary CT angiography was performed on a dual-source CT (DSCT) scanner (Somatom Definition, Siemens Healthcare, Forchheim, Germany). The scanning parameters were as follows: tube voltage 120 kV; tube current 320–400 mAs; detector collimation 2 × 32 × 0.6 mm; pitch 0.2–0.5; tube rotation time 330 ms; temporal resolution 83 ms. Patients with heart rates ≥ 65 beats/min were administered metoprolol one hour before the scan and sublingual nitroglycerin immediately prior to the CT scan. Retrospective ECG-gated CCTA was applied to the scan. Bolus tracking technique was used for all CCTA scans and the triggering threshold was set to a CT attenuation of 100 HU in the ascending aorta. The scan was obtained with intravenous injection of 40–70 ml iomeprol (400 mgI/ml, Iomeron 400; Bracco Imaging, Milan, Italy) at a flow rate of 4–6 ml/s, followed by 30 ml saline flush at the same flow rate. CCTA scan was acquired from 2 cm below the level of the tracheal bifurcation to 1–2 cm below the level of the diaphragm. Image data were routinely reconstructed in 35–45% and 65–75% of the R-R interval, with a slice thickness of 0.75 mm, slice increment of 0.5 mm, and a medium to smooth convolution kernel of B26f.

### Invasive coronary angiography

Invasive coronary angiography (CAG) was performed with standard practices and projections. The maximal diameter stenosis of each major coronary artery was assessed by an experienced observer who was blinded to the results of CCTA. The CAG-determined lumen narrowing ≥ 50% was defined as significant coronary stenosis. The degree of left coronary artery stenosis was defined by the maximal stenosis of proximal or middle parts of LAD or LCx. The degree of coronary stenosis severity was classified as mild (< 50%), moderate (50–69%), severe (70–99%) and total occlusion (100%).

### Reconstruction and analysis of CT images

**Fig 1:**
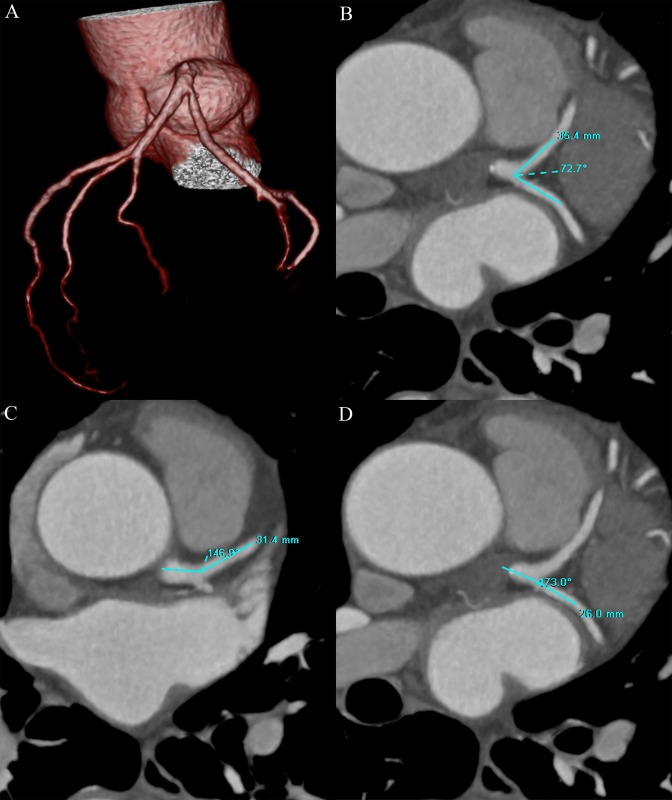
Volume rendering (VR) image of the coronary artery tree showed the left coronary bifurcation angles (A). The left coronary bifurcation angles of LAD-LCx (B), LM-LAD (C) and LM-LCx (D) were 72.7°, 146.9° and 173.0° on MPR images in a 62 year-old man with mild LAD stenosis. LM = left main coronary artery; LAD = left anterior descending artery; LCx = left circumflex artery; MPR = multiplanar reformation.

**Fig 2:**
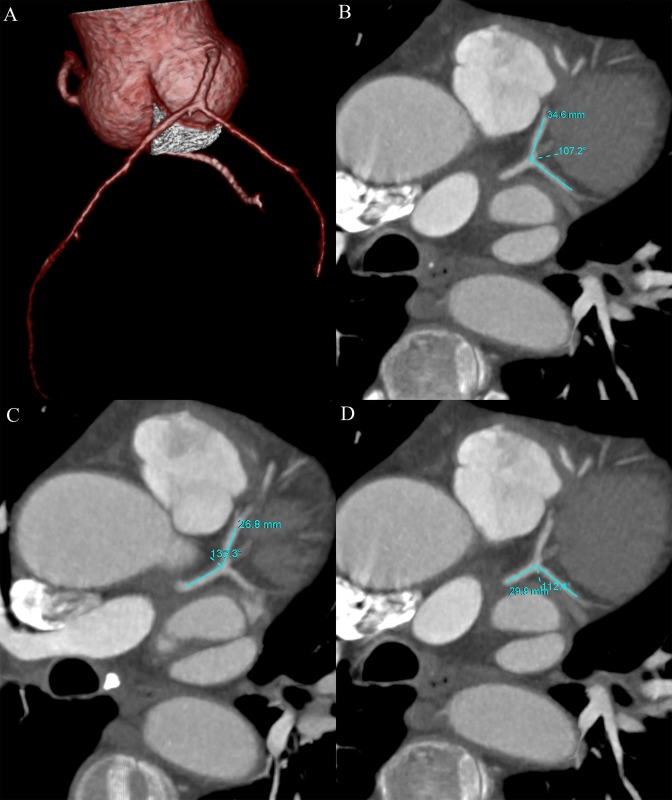
The left coronary bifurcation angles were showed by VR image (A). The left coronary bifurcation angles of LAD-LCx (B), LM-LAD (C) and LM-LCx (D) were 107.2°, 137.3° and 112.4° on MPR images in a 60 year-old man with proximal LAD stenosis of 55%. VR = volume rendering; LM = left main coronary artery; LAD = left anterior descending artery; LCx = left circumflex artery; MPR = multiplanar reformation.

All CT images were transferred to a dedicated workstation (Vitrea, version 6.0, Vital Images Inc., Toshiba Medical Systems, Japan) for further analysis by two experienced readers (with more than 5 years experience in cardiac CT imaging) who were blinded to the results of CAG. Multiplanar reconstruction (MPR) was performed exactly in the plane of LM, LAD and LCx vessels at the left coronary bifurcation. The bifurcation angles of the LAD-LCx, LM-LAD and LM-LCx were calculated after identifying the centerline vectors along the course of the LM, LAD and LCx in the MPR images (Figs 1 and 2). The bifurcation angles were measured in both systole and diastole, and were compared between the two phases. To examine intra-observer variability, one observer (Y.C.) measured the bifurcation angles of the LAD-LCx two times. To evaluate inter-observer variability, another observer (J.Y.) measured the bifurcation angles of the LAD-LCx using the same method and compared the measurements with the first observer.

**Fig 3:**
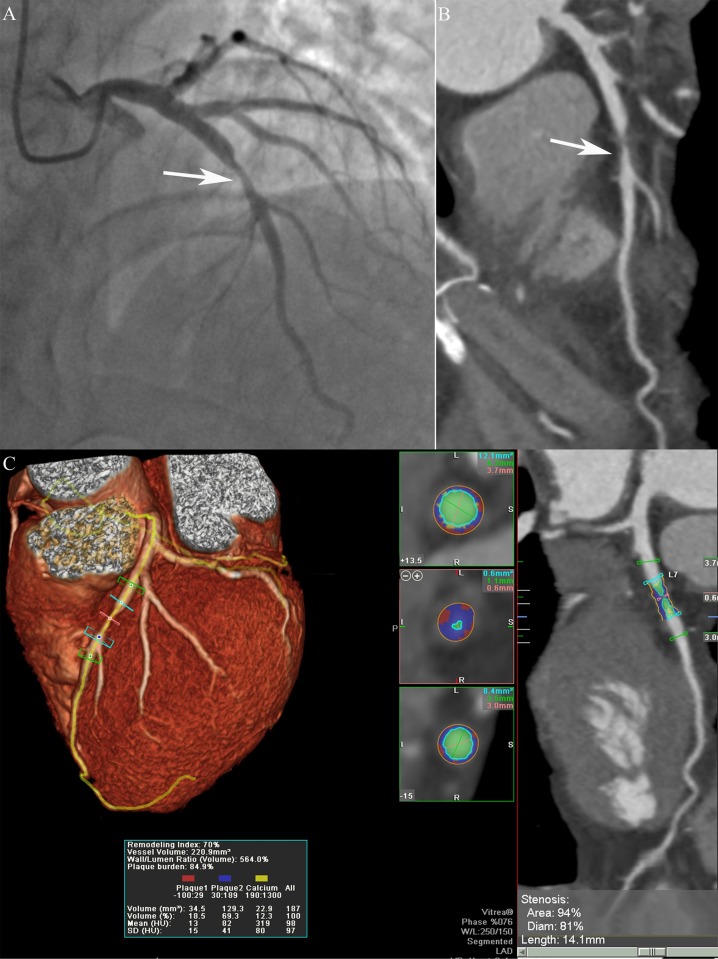
Invasive coronary angiography showed a serious lesion (arrow) in the proximal LAD with a stenosis of 80% (A). The CPR image showed a non-calcified plaque (arrow) in the proximal LAD (B). The semi-automatic plaque analysis software showed the lipid (red), fibrous (blue) and calcified (yellow) plaque through tracking of LAD (C). The CCTA plaque parameters were automatically measured and displayed in the images. LAD = left anterior descending artery; CPR = curved planar reformation; CCTA = coronary computed tomography angiography.

The dedicated software tool SurePlaque (Vitrea, version 6.0, Vital Images Inc., Toshiba Medical Systems, Japan) was used for plaque analysis, as previously described [13, 14]. The centerlines of the vessels were automatically tracked based on the opacification of the lumen. The plaque analysis software can detect the vessel lumens and plaques in CT images (Fig 3). The percentage of diameter or area stenosis was defined as maximal stenosis diameter or area divided by the average diameter or area of proximal and distal normal reference sites. The lesion length was defined as the length of the diseased vessels measured by the software. The minimal luminal diameter (MLD) and minimal luminal area (MLA) were automatically calculated by the plaque analysis software. The plaque burden was defined by the ratio of the plaque volume to the vessel volume (plaque volume×100%/vessel volume). The remodeling index was defined by the ratio of the vessel cross-sectional area at the site of maximal stenosis to the average of proximal and distal normal reference point cross-sectional areas [15]. Plaque volume was calculated using the sum of the contiguous voxels between the vessel and lumen contour of the lesion. According to previous studies [13, 14], different CT density values indicated different plaque components which were showed with different colors (Fig 3). The low CT value of -100 to 29 HU was defined as lipid (red) composition of the plaque. The CT value between 30 to 189 HU, and 350 to 1,000 HU were perceived as fibrous (blue) and calcified (yellow) compositions, respectively. The CT value between 190 and 349 HU was considered as lumen density (green). The final measurements could be manual corrected, if necessary.

Based on the volume of calcium composition, the plaques were classified into three types: non-calcified plaque (calcified component < 30% of the plaque volume), calcified plaque (calcified component > 70% of the plaque volume), and mixed plaque (30–70%). For ease of analysis, mixed plaques and calcified plaques were combined into the calcified plaque category. The left coronary artery was classified as non-calcified lesions if the stenotic portions of LAD and LCx were both non-calcified, and as calcified lesions if at least one vessel of LAD and LCx was calcified.

### Statistical analysis

Continuous variables are expressed as the mean ± standard deviation (SD), while categorical variables as frequency or percentage. Continuous variables were examined using unpaired t test for normal distribution or using the Mann-Whitney U test for non-normal distribution. Categorical variables were compared using Pearson’s χ^2^ or Fisher exact test. We used Kruskal–Wallis test to compare bifurcation angle of LAD-LCx among multiple groups (stenosis < 50% group, stenosis 50–69% group and stenosis 70–100% group), with adjusted P values from pairwise post hoc comparisons using the Dunn- Bonferroni test. The correlations between left coronary bifurcation angles and left coronary stenosis severity were examined by Pearson correlation coefficient. Multivariate logistic regression analysis odds ratio (OR) value with 95% confidence intervals (CI) was used to evaluate independent predictors of significant coronary stenosis and the final variable selection of independent predictors was performed with a backward stepping algorithm (S1 File). Receiver operating characteristics (ROC) curve analysis was performed to evaluate the diagnostic performance of left coronary bifurcation angles and plaque characteristics for detection of coronary stenosis. The optimal cutoff of the calculated variables was assessed using the highest Youden’s index values. Using the optimal cutoff, the sensitivity, specificity, positive predictive value (PPV) and negative predictive value (NPV) were assessed and described with a corresponding 95% CI. Inter-observer and intra-observer variability were determined by intraclass correlation coefficient (ICC) with the 95% CI. For all tests, a two-sided *P*< 0.05 was considered to be statistically significant. Statistical analyses were performed by IBM SPSS Statistics 19 (IBM Corp., Armon, NY) and MedCalc 16.2.0 (MedCalc Software, Mariakerke, Belgium).

## Results

### Clinical characteristics

A total of 119 patients undergoing CCTA and invasive CAG within three months were retrospectively reviewed in this study. Of these, 13 (10.9%) patients were excluded, two due to poor CT image quality, two due to congenital coronary artery anomalies and nine because of lacking multiple-phase image data. In the 106 patients, eight vessels with a proximal diameter < 2 mm and 78 normal vessels were excluded. Finally, 318 coronary bifurcation angles (106 LAD-LCx, 106 LM-LAD and 106 LM-LCx) and 126 coronary vessels (79 LAD and 47 LCx) were enrolled for analysis (S2 and S3 Files). Of these vessels, 86 coronary vessels (58 LAD and 28 LCx) with ≥ 50% luminal narrowing were detected by CAG. The mean distance of the throat of each lesion from the LM bifurcation were 10.1±7.8mm (rang: 0–32.1mm) and 8.3±9.2mm (rang: 0–23.0mm) for LAD and LCx, respectively. The baseline characteristics of the study population are summarized in Table 1.

**Table 1:** Baseline characteristics of the study population.

Characteristics	Value, n = 106
**Age (mean+SD), years**	59.9 ± 10.8
**Male, n (%)**	75 (70.8)
**BMI (mean+SD), kg/m**^**2**^	24.2 ± 3.4
**Risk factors**	
**Hypertension, n (%)**	62 (58.5)
**Hyperlipidemia, n (%)**	29 (27.4)
**Diabetes, n (%)**	27 (25.5)
**Currently smoking, n (%)**	41 (38.7)
**Currently drinking, n (%)**	28 (26.4)
**Family history of CAD, n (%)**	20 (18.9)
**Percutaneous coronary intervention, n (%)**	23 (21.7)
**Previous myocardial infarction, n (%)**	5 (4.7)
**Angina at presentation**	
**Unstable angina, n (%)**	61 (57.5)
**Stable angina, n (%)**	8 (7.5)
**Coronary artery stenosis (CAG ≥ 50%)**	86
**LAD, n (%)**	58 (67.4)
**LCx, n (%)**	28 (32.6)

BMI = body mass index; CAD = coronary artery disease; CAG = coronary angiography; LAD = left anterior descending artery; LCx = left circumflex artery.

### Relationship between left coronary bifurcation angles and different levels of left coronary stenosis severity by CAG

**Fig 4:**
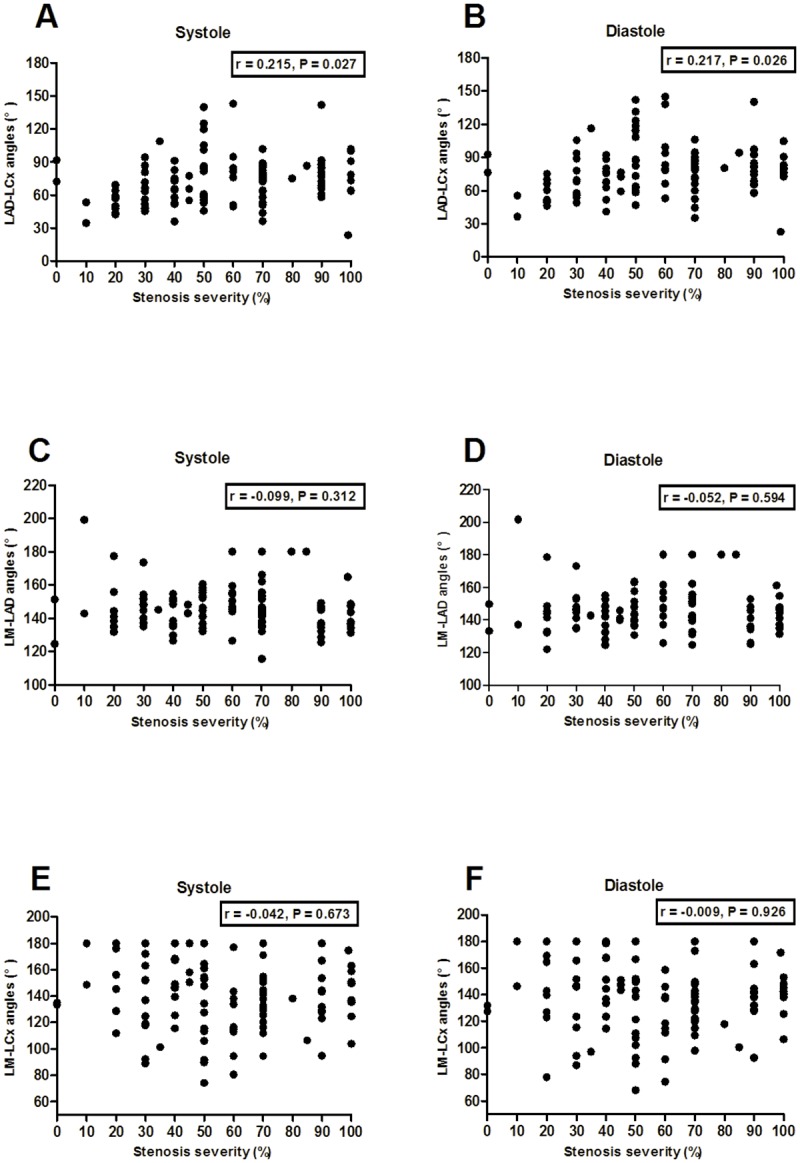
There was mild correlation between the bifurcation angle of LAD-LCx and left coronary stenosis severity at systole (A) and diastole (B). The bifurcation angle of LM-LAD and LM-LCx showed no significant association with left coronary stenosis severity at both systole (C, E) and diastole (D, F).

The bifurcation angles of LAD-LCx, LM-LAD and LM-LCx in different degrees of left coronary artery stenosis severity are listed in Table 2. The bifurcation angle of LAD-LCx in stenosis < 50% was significantly smaller than that in stenosis ≥ 50%, from 68.3° ± 18.0° for stenosis < 50% to 91.3° ± 29.8° for stenosis of 50–69%, 80.0°±19.2° for stenosis of 70–100% at diastole (*P* = 0.001). A similar trend was seen at systole (*P* = 0.001). However, the bifurcation angles of LM-LAD and LM-LCx were comparable for different levels of left coronary artery stenosis severity at diastole. It showed mild correlation between the bifurcation angle of LAD-LCx and left coronary stenosis severity at systole (r = 0.215, *P* = 0.027) and diastole (r = 0.217, *P* = 0.026) (Fig 4). The bifurcation angle of LM-LAD and LM-LCx showed no significant correlation with left coronary stenosis severity at both systole and diastole (Fig 4). Table 3 demonstrates that the bifurcation angles of LAD-LCx and LM-LCx were significantly larger at diastole than systole (P < 0.001).

**Table 2:** Left coronary bifurcation angle in different degrees of left coronary stenosis severity determined by CAG.

Left coronary bifurcation angle (°)	Stenosis < 50% (n = 37)	Stenosis 50–69% (n = 24)	Stenosis 70–100% (n = 45)	P value
**Systole**				
**LAD-LCx**	63.4 ± 17.3	86.5 ± 30.7	75.0±19.0	0.001
**LM-LAD**	146.5 ± 14.0	147.7 ± 11.9	144.9±13.6	0.685
**LM-LCx**	145.9 ± 25.9	128.3 ± 30.0	141.4 ± 22.7	0.031
**Diastole**				
**LAD-LCx**	68.3 ± 18.0	91.3 ± 29.8	80.0±19.2	0.001
**LM-LAD**	145.2 ± 14.7	147.3 ± 12.1	145.5 ± 13.2	0.822
**LM-LCx**	140.5 ± 27.0	125.6 ± 29.8	137.1 ± 20.6	0.067

Values are presented as mean ± SD.

*P* = 0.014 and *P* = 0.002 for comparison of LAD-LCx between stenosis < 50% and stenosis 50–69% at systole and diastole, respectively

*P* = 0.003 and *P* = 0.006 for comparison of LAD-LCx between stenosis < 50% and stenosis 70–100% at systole and diastole, respectively

*P* = 0.985 and *P* = 1.000 for comparison of LAD-LCx between stenosis 50–69% and stenosis 70–100% at systole and diastole, respectively

LM = left main coronary artery; LAD = left anterior descending artery; LCx = left circumflex artery.

**Table 3:** Comparison of left coronary bifurcation angles between systole and diastole.

Left coronary bifurcation angle(°)	Total (n = 106)			CAG stenosis < 50% (n = 37)			CAG stenosis ≥ 50% (n = 69)		
	Systole	Diastole	P value	Systole	Diastole	P value	Systole	Diastole	P value
**LAD-LCx**	73.6 ± 23.1	78.4 ± 23.1	< 0.001	63.4 ± 17.3	68.3 ± 18.0	< 0.001	79.0 ± 24.1	83.8 ± 23.8	< 0.001
**LM-LAD**	146.1 ± 13.3	145.8 ± 13.4	0.605	146.5 ± 14.0	145.2 ± 14.7	0.199	145.8 ± 13.0	146.1 ± 12.8	0.646
**LM-LCx**	140.0 ± 26.2	135.7 ± 25.6	< 0.001	145.9 ± 25.9	140.5 ± 27.0	0.001	136.9 ± 26.0	133.1 ± 24.6	< 0.001

Values are presented as mean ± SD.

CAG = coronary angiography; LM = left main coronary artery; LAD = left anterior descending artery; LCx = left circumflex artery.

### Relationship between left coronary bifurcation angles and plaque characteristics

The left coronary bifurcation angles of LAD-LCx, LM-LAD and LM-LCx in different plaque types at systole and diastole are shown in Table 4. Among the 106 patients, 102 with 58 calcified and 44 non-calcified lesions in the left coronary artery were analyzed. As compared to the left coronary calcified group, the bifurcation angles of LAD-LCx in non-calcified lesions were significantly wider at systole and diastole (79.5° ± 26.6° vs. 69.8° ± 19.2°, *P* = 0.034 and 84.3° ± 25.7° vs. 74.8° ± 20.1°, *P* = 0.037, respectively). In the stenosis ≥ 50% group, the same trend was observed (*P* < 0.05). However, there was no significant difference in stenosis < 50% (*P* > 0.05).

**Table 4:** Comparison of left coronary bifurcation angles between the non-calcified plaque and calcified plaque groups.

		Systole			Diastole		
Left coronary bifurcation angle (°)	n	Non-calcified plaque	Calcified plaque	P value	Non-calcified plaque	Calcified plaque	P value
**Total**	102	44	58		44	58	
**LAD-LCx**		79.5 ± 26.6	69.8 ± 19.2	0.034	84.3 ± 25.7	74.8 ± 20.1	0.037
**LM-LAD**		145.6 ± 11.5	145.9 ± 13.0	0.891	144.4 ± 12.4	146.2 ± 12.5	0.464
**LM-LCx**		137.3 ± 29.7	141.5 ± 23.7	0.431	132.5 ± 28.4	137.4 ± 23.4	0.341
**CAG stenosis ≥ 50%**	69	29	40		29	40	
**LAD-LCx**		87.9 ± 26.6	72.5 ± 20.2	0.008	92.2 ± 24.9	77.7 ± 21.3	0.011
**LM-LAD**		146.1 ± 13.4	145.6 ± 13.0	0.877	145.7 ± 13.5	146.4 ± 12.4	0.823
**LM-LCx**		130.7 ± 26.8	141.3 ± 24.9	0.096	125.7 ± 24.4	138.5 ± 23.6	0.032
**CAG stenosis < 50%**	33	15	18		15	18	
**LAD-LCx**		63.3 ± 18.5	63.7 ± 15.7	0.950	69.1 ± 20.1	68.3 ± 15.7	0.905
**LM-LAD**		144.4 ± 7.0	146.5 ± 13.3	0.598	141.8 ± 9.8	145.8 ± 12.9	0.340
**LM-LCx**		150.0 ± 31.9	141.8 ± 21.6	0.392	145.6 ± 31.6	134.9 ± 23.5	0.276

Values are presented as mean ± SD.

CAG = coronary angiography; LM = left main coronary artery; LAD = left anterior descending artery; LCx = left circumflex artery.

### Diagnostic performance of bifurcation angles of LAD-LCx in left coronary stenosis

**Fig 5:**
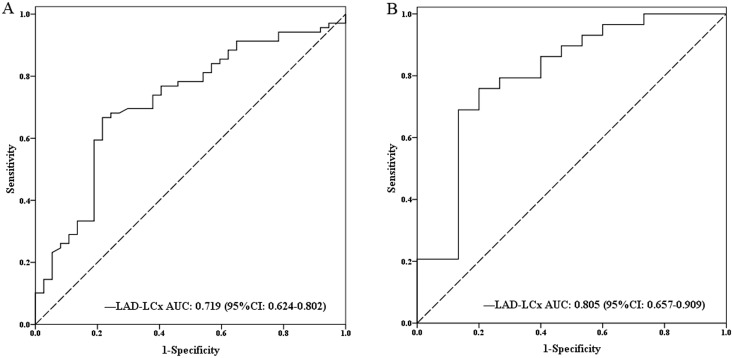
Receiver operating characteristic (ROC) curve analysis showed bifurcation angle of LAD-LCx for prediction of significant left coronary artery stenosis in total (A) and non-calcified (B) left coronary artery. AUC = area under the receiver-operating characteristics curve; CI = confidence interval; LAD = left anterior descending artery; LCx = left circumflex artery.

The mean bifurcation angle of LAD-LCx was 78.4 ± 23.1° (range: 22.6°-144.6°) among all the patients. Table 5 showed hyperlipidemia and larger bifurcation angles of LAD-LCx were associated with higher incidence of significant left coronary stenosis. In the multivariate logistic regression analysis, the bifurcation angles of LAD-LCx were independent predictors for identifying significant left coronary stenosis (OR = 1.423, *P* = 0.002, 95% CI 1.140–1.777) (Table 5). In ROC curve analysis, the optimal cutoff value of LAD-LCx was 78° for classifying significant left coronary stenosis at diastole. It showed an area under the curve (AUC) of 0.719 (*P* < 0.001) for LAD-LCx in total vessels and 0.805 (*P* < 0.001) for LAD-LCx in non-calcified vessels (Fig 5). At a threshold of ≥ 78°, LAD-LCx predicted significant left coronary stenosis with a sensitivity of 66.7% (54.3–77.6%), specificity of 78.4% (61.8–90.2%), PPV of 85.2% (72.9–93.4%) and NPV of 55.8% (41.3–69.5%), respectively. (Table 6). The sensitivity, specificity, PPV and NPV of CCTA diameter stenosis were 85.5% (75.0–92.8%), 78.4% (61.8–90.2%), 88.1% (77.8–94.7%), and 74.4% (57.9–87.0%), respectively. The addition of bifurcation angle of LAD-LCx to the diameter stenosis derived from CCTA showed the sensitivity of 82.6% (71.6–90.7%), specificity of 89.2% (74.6–97.0%), PPV of 93.4% (84.1–98.2%), and NPV of 73.3% (58.1–85.4%), respectively. The addition of bifurcation angle of LAD-LCx to CCTA diameter stenosis improved the specificity and PPV.

**Table 5:** Multivariate logistic regression analysis of CCTA parameters for predicting coronary stenosis by CAG.

Variables	B	SE	χ^2^ value	p value	OR (95% CI)
**Hyperlipidemia**	1.267	0.570	4.942	0.026	3.551 (1.162–10.854)
**LAD-LCx (per 10°)**	0.353	0.113	9.715	0.002	1.423 (1.140–1.777)
**Diameter stenosis**	0.082	0.018	19.685	< 0.001	1.085 (1.047–1.125)
**Lipid plaque volume (per 10mm**^**3**^**)**	0.463	0.139	11.125	0.001	1.589 (1.210–2.086)

OR = odds ratio; CI = confidence interval; LAD = left anterior descending artery; LCx = left circumflex artery.

**Table 6:** Diagnostic performance of left coronary bifurcation angles and plaque characteristics for identifying CAG-determined coronary stenosis.

Cutoff value	AUC	Sensitivity (%)	Specificity (%)	PPV (%)	NPV (%)
**Total vessels**					
**LAD-LCx ≥ 78°**	0.719 (0.624–0.802)	66.7 (54.3–77.6)	78.4 (61.8–90.2)	85.2 (72.9–93.4)	55.8 (41.3–69.5)
**Non-calcified vessels**					
**LAD-LCx ≥ 78°**	0.805 (0.657–0.909)	75.9 (56.5–89.7)	80.0 (51.9–95.7)	88.0 (68.8–97.5)	63.2 (38.4–83.7)
**Diameter stenosis ≥ 44%**	0.854 (0.780–0.911)	73.3 (62.6–82.2)	87.5 (73.2–95.8)	92.6 (83.7–97.6)	60.3 (46.6–73.0)
**Lipid plaque volume ≥ 33 mm**^**3**^	0.782 (0.700–0.851)	65.1 (54.1–75.1)	82.5 (67.2–92.7)	88.9 (78.4–95.4)	52.4 (39.4–65.1)
**Diameter stenosis + lipid plaque volume**	0.900 (0.833–0.946)	83.7 (74.2–90.8)	85.0 (70.2–94.3)	92.3 (84.0–97.1)	70.8 (55.9–83.0)

AUC = area under the receiver-operating characteristics curve; PPV = positive predictive value; NPV = negative predictive value; LAD = left anterior descending artery; LCx = left circumflex artery.

As shown in Table 7, the patient characteristics and risk factors were comparable between the different bifurcation angles of LAD-LCx, using 78° as the cutoff. The maximal diameter stenosis, maximal area stenosis and total plaque burden of the left coronary artery demonstrated significant differences between bifurcation angles of LAD-LCx ≥ 78° and <78° (58.1± 26.6% vs. 39.4 ± 28.3%, *P* < 0.001; 65.3 ± 28.9% vs. 45.0 ± 32.0%, *P* < 0.001; 93.8 ± 41.4% vs. 72.7 ± 50.3%, *P* = 0.020, respectively). However, the CCTA parameters of total lesion length, total lipid plaque volume, total fibrous plaque volume, total calcified plaque volume and total plaque volume of the left coronary artery were similar between the two groups. The results are listed in Table 7.

**Table 7:** Clinical data analysis of different bifurcation angles.

	LAD-LCx ≥ 78 (n = 54)	LAD-LCx < 78 (n = 52)	P value
**Patient characteristics**			
**Age (years)**	58.0 ± 12.0	61.9 ± 9.1	0.061
**Male, n (%)**	42 (77.8)	33 (63.5)	0.105
**BMI (kg/m**^**2**^**)**	24.4 ± 3.5	24.0 ± 3.4	0.515
**Risk factors**			
**Hypertension, n (%)**	29 (53.7)	33 (63.5)	0.308
**Dyslipidemia, n (%)**	17 (31.5)	12(23.1)	0.332
**Diabetes, n (%)**	14 (25.9)	13 (25.0)	0.913
**Currently smoking, n (%)**	24 (44.4)	17 (32.7)	0.214
**Currently drinking, n (%)**	18 (33.3)	10 (19.2)	0.100
**Family history of CAD, n (%)**	10 (18.5)	10 (19.2)	0.925
**Left coronary CCTA parameters**			
**Maximal diameter stenosis (%)**	58.1± 26.6	39.4 ± 28.3	< 0.001
**Maximal area stenosis (%)**	65.3 ± 28.9	45.0 ± 32.0	< 0.001
**Total lesion length (mm)**	30.8 ± 25.2	25.5 ± 25.4	0.286
**Total plaque burden (%)**	93.8 ± 41.4	72.7 ± 50.3	0.020
**Lipid plaque volume (mm**^**3**^**)**	64.6 ± 51.9	52.6 ± 52.4	0.240
**Fibrous plaque volume (mm**^**3**^**)**	204.1 ± 161.0	167.6 ± 155.0	0.238
**Calcified plaque volume (mm**^**3**^**)**	91.2 ± 171.3	54.7 ± 93.8	0.326
**Total plaque volume (mm**^**3**^**)**	359.8 ± 345.5	274.6 ± 278.9	0.198

Continuous data are presented as mean ± SD.

BMI = body mass index; CAD = coronary artery disease; LAD = left anterior descending artery; LCx = left circumflex artery.

### Relationship between morphological plaque characteristics and coronary stenosis by CAG

Table 8 showed the results of quantitative plaque analysis in coronary stenosis < 50% vs. stenosis ≥ 50%. Total plaque volume, lipid and fibrous plaque volumes, lumen diameter stenosis, lumen area stenosis, lesion length and plaque burden were significantly larger or longer in the group with stenosis ≥ 50% than that in the stenosis < 50% group, while MLD and MLA were smaller (all *P*< 0.05). However, calcified plaque volume and remodeling index were comparable between the two groups.

**Table 8:** Per-vessel CCTA characteristics in coronary stenosis by CAG.

CCTA parameters	Stenosis < 50% (n = 40)	Stenosis ≥ 50% (n = 86)	P value
**Diameter stenosis (%)**	31.5 ± 13.6	58.6 ± 20.9	< 0.001
**Area stenosis (%)**	35.0 ± 19.8	64.5 ± 25.1	< 0.001
**Lesion length (mm)**	12.0 ± 6.1	24.3 ± 14.4	< 0.001
**MLD (mm)**	2.1 ± 0.6	1.3 ± 0.8	< 0.001
**MLA (mm**^**2**^**)**	6.0 ± 3.1	3.2 ± 2.9	< 0.001
**Plaque burden (%)**	60.0 ± 8.3	64.6 ± 9.4	0.010
**Remodeling index**	1.1 ± 0.2	1.0 ± 0.5	0.365
**Lipid plaque volume (mm**^**3**^**)**	24.6 ± 16.7	50.5 ± 30.4	< 0.001
**Lipid plaque volume (mm**^**3**^**)**	84.0 ± 46.2	160.4 ± 91.7	< 0.001
**Calcified plaque volume (mm**^**3**^**)**	24.9 ± 40.4	45.1 ± 64.4	0.331
**Total plaque volume (mm**^**3**^**)**	133.0 ± 88.3	256.0 ± 158.0	< 0.001

Values are expressed as mean ± SD.

CCTA = coronary computed tomography angiography; MLD = minimum lumen diameter; MLA = minimum lumen area.

### Diagnostic performance of plaque characteristics in coronary stenosis

**Fig 6:**
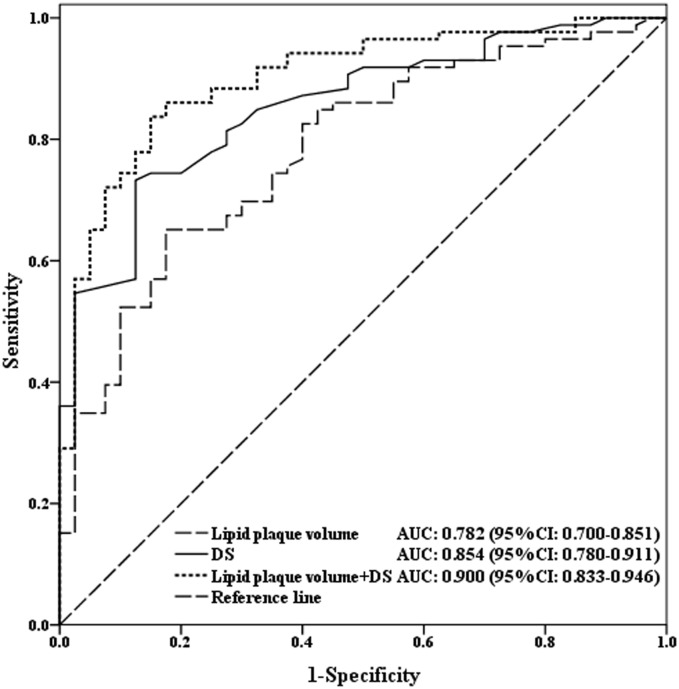
Receiver operating characteristic (ROC) curve analysis showed diameter stenosis (DS), lipid plaque volume and diameter stenosis + lipid plaque volume for identifying significant coronary artery stenosis. AUC = area under the receiver-operating characteristics curve; CI = confidence interval.

The lipid plaque volume was an independent predictor for identifying significant coronary stenosis (OR = 1.589, *P* = 0.001, 95% CI 1.210–2.086) (Table 5). The AUC (95% CI) for discriminating CAG-determined stenosis ≥ 50% was 0.782 (0.700–0.851) for lipid plaque volume and 0.854 (0.780–0.911) for CCTA diameter stenosis (Fig 6). The addition of lipid plaque volume ≥ 33 mm^3^ to CCTA diameter stenosis provided incremental prediction of significant coronary stenosis (0.854 vs. 0.900, *P* = 0.045). The sensitivity, specificity, PPV and NPV of lipid plaque volume cutoff ≥ 33mm^3^ were 65.1% (54.1–75.1%), 82.5% (67.2–92.7%), 88.9% (78.4–95.4%), and 52.4% (39.4–65.1%), respectively. The addition of lipid plaque volume to the diameter stenosis derived from CCTA improved the sensitivity to 83.7% (74.2–90.8%), specificity to 85.0% (70.2–94.3%), PPV to 92.3% (84.0–97.1%) and NPV to 70.8% (55.9–83.0%) (Table 6).

### Repeatability analysis

The inter-observer and intra-observer agreements were analyzed in the 106 patients. The ICCs with the 95% CI for inter-observer and intra-observer variability were 0.963 (95%CI 0.946–0.975) and 0.993 (95%CI 0.989–0.995) for measurement of bifurcation angles of LAD-LCx at diastole. The mean time was 1.8 ± 0.7 min for measurement of bifurcation angles of LAD-LCx and was 3.4 ± 0.9 min for measurement of bifurcation angles of LAD-LCx, LM-LAD and LM-LCx at both systole and diastole.

## Discussion

In the present study, we demonstrated a direct relationship between left coronary bifurcation angles and left coronary stenosis severity. The results showed that wider bifurcation angle of LAD-LCx was associated with significant left coronary stenosis, greater plaque burden, and non-calcified plaques. It also confirmed that the bifurcation angle of LAD-LCx and lipid plaque volume were independent predictors for significant coronary stenosis and lipid plaque volume could improve the diagnostic performance of CCTA diameter stenosis for the prediction of obstructive CAD.

Our results demonstrated that the left coronary bifurcation angles of LAD-LCx were related to CAG-determined left coronary stenosis severity, which was not discussed in previous studies. The possible mechanisms responsible for these results may be the hemodynamic factors. The 3D geometry of bifurcation angle of LAD-LCx changed the coronary hemodynamic environment and induced the formation of atherosclerosis. Larger bifurcation angles produced greater turbulence and hemodynamic impact on the blood flow, which was subsequently associated with CAD. By using CFD in 3D reconstruction of the left main coronary artery and bifurcation, they found that low endothelial shear stress, occurring in disturbed flow patterns, was related to plaque location and thickness in the bifurcations [16]. Additionally, the local hemodynamic flow patterns have an important effect on the development of coronary eccentric plaques at earlier and advanced stage of atherosclerosis [17]. Papadopoulou et al. evaluated the distribution and composition of plaques between CCTA and intravascular ultrasound-virtual histology (IVUS-VH), and showed that wider bifurcation angles were related to the presence of high-risk and non-calcified plaques or CAD [18]. Our results were in agreement with the above studies. Additionally, our data suggested that bifurcation angle of LAD-LCx showed mild correlation with left coronary stenosis severity. Wider bifurcation angles of LAD-LCx could predict significant coronary stenosis with moderate sensitivity and NPV, and high specificity and PPV.

The causes of coronary artery atherosclerosis are multifactorial and identification of these risk factors could allow earlier detection and prevention of the coronary artery disease. The bifurcation angle of LAD-LCx was one of the many risk factors which were related to atherosclerosis. The measurement of bifurcation angles of LAD-LCx could be a useful tool for evaluation of left coronary stenosis and plaque burden. This study may provide a clinically useful approach to exploring the possible role for this branching angle as a risk indicator of earlier development and progression of plaque in the coronary bifurcation. It might be an indicator for commencing treatment prior to the onset of plaque formation. In addition, it took only a few extra minutes to measure the angulations for every patient. Therefore, this quick method can be easily applied to clinical routine.

DSCT is a reliable noninvasive modality for detecting obstructive CAD with high temporal and spatial resolutions, irrespective of the heart rate [19]. Therefore, DSCT can reconstruct robust image quality at both systole and diastole. In the present study, the bifurcation angles were accurately measured at the two phases. Our data suggested that the bifurcation angles of LAD-LCx and LM-LCx at diastole were significantly larger than that at systole, which may be due to the cardiac motion during the cardiac cycle. Accordingly, DSCT may be able to objectively measure the left coronary bifurcation angles. In this study, a bifurcation angle of 78° was calculated as a cutoff value to identify significant coronary stenosis, which is mild smaller than the 80° reported by previous studies due to the smaller physical build of Asia people. Bifurcation angulations are known to play a vital role in bifurcation intervention. The anatomical information on specific bifurcation angles of the left coronary artery may be useful for the selection and implementation of bifurcation stenting strategies and treatment of complex bifurcation lesions [20, 21].

This study also confirmed that lipid plaque volume was an independent predictor for obstructive CAD and could improve the diagnostic performance of CCTA in coronary stenosis. Previous studies showed that low density non-calcified plaque and non-calcified plaque volumes or burden could predict lesion-specific ischemia [22–24]. Low CT value (< 30 HU), likely to be lipid-rich plaques that may tend to rupture was related to major adverse cardiac events [25]. The quantification of CT values of atherosclerotic plaques could help to differentiate lipid, fibrous and calcified plaques. In this study, with CAG as the reference standard, the addition of lipid plaque volume to diameter stenosis on CCTA increased the diagnostic value for determination of obstructive CAD.

The study had several limitations. First, it was a single-center study with 106 patients, and larger multi-center studies with more participants are required to verify the results. Second, this study was performed with invasive CAG as a reference standard, and the relationship between bifurcation angles and lesion-specific ischemia by FFR needs to be further explored.

## Conclusions

This study evaluated the diagnostic performance of left coronary bifurcation angles and plaque characteristics for prediction of coronary stenosis. Results showed that the bifurcation angle of LAD-LCx may predict significant left coronary stenosis. Wider bifurcation angle of LAD-LCx was related to larger plaque burden and non-calcified lesions. The addition of lipid plaque volume to diameter stenosis on CCTA increased the diagnostic performance for the determination of obstructive CAD.

## Supporting information

S1 File Multivariate logistic regression analysis. The variables entered in the multivariable model were showed in details.
(PDF)

S2 File Patient clinical characteristics. A list of the baseline characteristics and left coronary bifurcation angles of the study population.
(XLSX)

S3 File Patient CCTA characteristics. A list of per-vessel CCTA parameters of the study population.
(XLSX)
