# Supplementary material for: Quantification of left coronary bifurcation angles and plaques by coronary computed tomography angiography for prediction of significant coronary stenosis: A preliminary study with dual-source CT
Source: PLoS One. 2017 Mar 27;12(3):e0174352. doi: 10.1371/journal.pone.0174352 (PMC5367806; doi:10.1371/journal.pone.0174352)
Supplement: S1 File — The variables entered in the multivariable model were showed in details. (PDF) [file pone.0174352.s001.pdf]

The variables entered in the multivariable model were showed in details. In the left coronary bifurcation angle analysis, the variables including age, sex, BMI, hypertension, hyperlipidemia, currently smoking, currently drinking, family history of CAD and bifurcation angles of LAD-LCx were entered in the multivariable model. In the plaque analysis, the variables including diameter stenosis, area stenosis, lesion length, MLD, MLA, plaque burden, lipid plaque volume, fibrous plaque volume and total plaque volume were entered in the multivariable model.
